# Updated Reference Genome Sequence and Annotation of *Mycobacterium bovis* AF2122/97

**DOI:** 10.1128/genomeA.00157-17

**Published:** 2017-04-06

**Authors:** Kerri M. Malone, Damien Farrell, Tod P. Stuber, Olga T. Schubert, Ruedi Aebersold, Suelee Robbe-Austerman, Stephen V. Gordon

**Affiliations:** a School of Veterinary Medicine, University College Dublin, Dublin, Ireland; b Diagnostic Bacteriology Laboratory, National Veterinary Services Laboratories, Ames, Iowa, USA; c Department of Biology, Institute of Molecular Systems Biology, ETH Zurich, Zurich, Switzerland; d School of Medicine, University College Dublin, Dublin, Ireland; e School of Biomolecular and Biomedical Science, University College Dublin, Dublin, Ireland; f UCD Conway Institute of Biomolecular and Biomedical Research, University College Dublin, Dublin, Ireland

## Abstract

We report here an update to the reference genome sequence of the bovine tuberculosis bacillus *Mycobacterium bovis* AF2122/97, generated using an integrative multiomics approach. The update includes 42 new coding sequences (CDSs), 14 modified annotations, 26 single-nucleotide polymorphism (SNP) corrections, and disclosure that the RD900 locus, previously described as absent from the genome, is in fact present.

## GENOME ANNOUNCEMENT

*Mycobacterium bovis*, the causative agent of bovine tuberculosis (bTB), is the most widely studied animal-adapted member of the *Mycobacterium tuberculosis* complex (MTBC). bTB exacts a tremendous global economic toll through productivity losses and disease control costs, while zoonotic transmission of *M. bovis* infection is a threat to human health (1–6).

*M. bovis* AF2122/97 was the first *M. bovis* strain to be sequenced and serves as the reference genome (7), and it was last updated in 2003. An updated reference *M. bovis* genome sequence will provide an essential resource for the tuberculosis (TB) research community and serve as a basis for comparative studies into animal- and human-adapted MTBC members.

To update the *M. bovis* AF2122/97 genome and annotation, a low-passage-number stock taken from the original *M. bovis* AF2122/97 seed stock was resequenced and reannotated using a combination of DNA sequencing, RNA sequencing, and proteomics data. All nucleic acid and protein samples were derived from exponentially grown cultures.

Short-read DNA sequencing libraries were prepared using the Nextera XT DNA library preparation kit (Illumina) and sequenced on the MiSeq system (Illumina), generating 250-bp paired-end reads that were trimmed using Sickle (*Q* > 30), with 60× reference coverage (8). For PacBio RSII sequencing, enzymatically extracted DNA was prepared using a large-insert library (6 kb to 8kb) size selection (9). Two single-molecule real-time (SMRT) cells were used for an output of 542,585,804 bases, a mean read length of 8,141, and 86× reference coverage. DNA sequencing data sets were analyzed using a combination of *de novo* assembly [short reads, SOAP*denovo* (10); long reads, Canu (11)] and nucleotide variant identification methods [short reads, Stampy, SAMtools, and VCFtools (12–14); long reads, Pilon (15); and MUMmer (16)]. This allowed both an update of the genome nucleotide sequence and the identification of genomic regions that had been misassembled, or missed entirely, in the original sequencing project. Reannotation of the *M. bovis* AF2122/97 genome was achieved by automatic annotation transfer from *M. tuberculosis* H37Rv (17) and a proteogenomic analysis using both *M. bovis* AF2122/97 shotgun tandem mass spectrometry (MS/MS), sequential window acquisition of all theoretical mass spectra (SWATH MS) data sets, and *M. tuberculosis* H37Rv SWATH MS data sets (18).

Overall, 26 single nucleotide polymorphisms were identified. Strikingly, the large sequence polymorphism RD900, originally described as being deleted from *M. bovis* 2122/97 (19), was found to be present; recombination between repeat structures flanking the RD900 locus in clones used for the original shotgun sequencing genome project may have led to the loss of RD900. Furthermore, 42 novel coding sequences were identified, while 14 existing annotations were modified.

### Accession number(s).

This whole-genome shotgun project had been deposited in DDBJ/ENA/GenBank under the accession no. LT708304. SWATH MS data can be found on PeptideAtlas under identifier PASS00932.
